# From Recurrent Syncope to Sudden Cardiac Death: Clinical Characteristics in a Chinese Patient Carrying a Plakophilin-2 Gene Mutation

**DOI:** 10.1155/2013/246891

**Published:** 2013-04-16

**Authors:** Wenling Liu, Xiaoliang Qiu, Wen Liu, Dayi Hu, Tiangang Zhu, Chunling Wang, Dominik Beer, Li Zhang

**Affiliations:** ^1^Peking University People's Hospital, Beijing 100044, China; ^2^China Meitan General Hospital, Beijing, China; ^3^Lankenau Medical Center, Lankenau Institute for Medical Research, Wynnewood, PA, USA

## Abstract

We report a case of Arrhythmogenic Right Ventricular Cardiomyopathy (ARVC) which illustrates the natural progression of disease in the absence of availability of an implanted cardiac defibrillator (ICD). Electrocardiograms and cardiac imaging show the progress of ARVC and these clinical milestones of disease are presented herein.

## 1. Background

ARVC is a largely inherited cardiomyopathy that affects mostly young otherwise healthy individuals and is associated with an increased risk of sudden death [1]. The morphological and arrhythmogenic substrate of ARVC predominantly affects the right ventricle (RV) [2] and is characterized by progressive myocardial atrophy with subsequent replacement by fatty and fibrous tissue [3]. Accordingly, the clinical process is also progressive [3, 4]. Here we review the entire progression of a young patient with ARVC from recurrent syncope to sudden cardiac death in the absence of ICD.

## 2. Case Presentation

**Figure 1:**
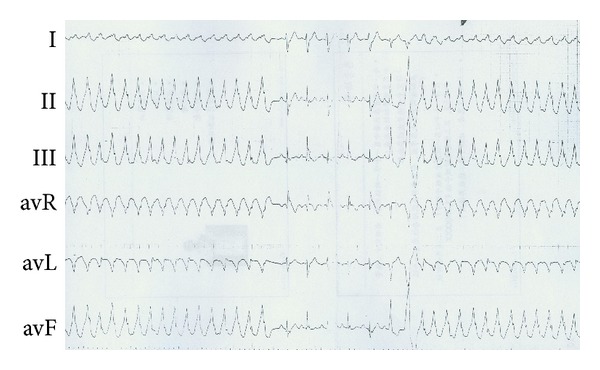
Body surface 12-lead ECG documented in 2005.

**Figure 2:**
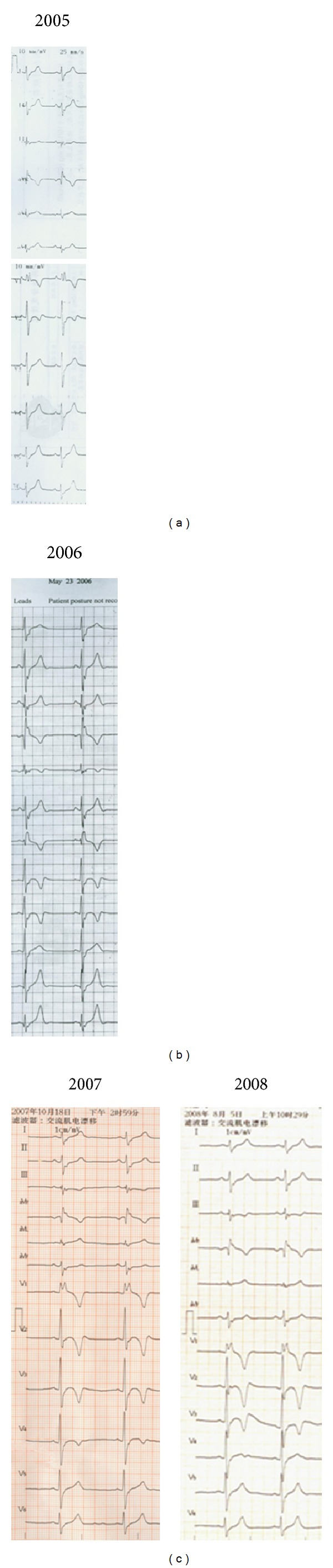
T-wave progression on ECG and (a) T-wave inversion in leads V1, V2 in 2005; (b) T-wave inversion in leads V1~V3 in 2006; (c) T-wave inversion in leads V1~V4 in 2007 and 2008.

**Figure 3:**
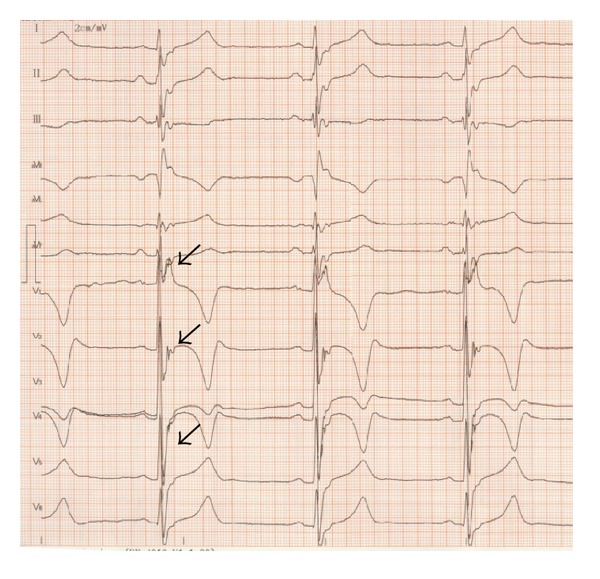
Body surface 12-lead ECG recording in 2 cm/mV and 50 mm/S. The arrows indicate epsilon waves, fractionated QRS complexes, and terminal S wave prolongation.

**Figure 4:**
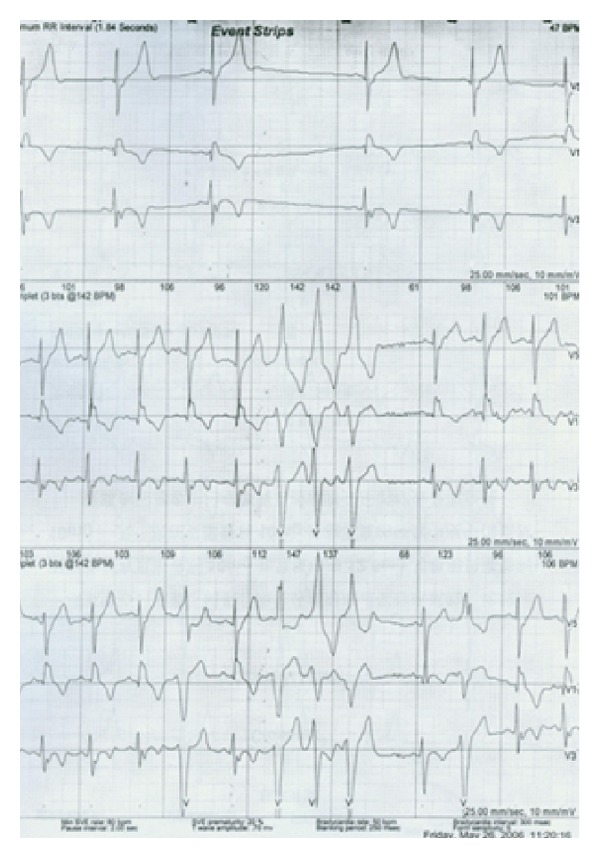
Holter ECG revealed frequent multifocal PVCs and nonsustained VT.

**Figure 5:**
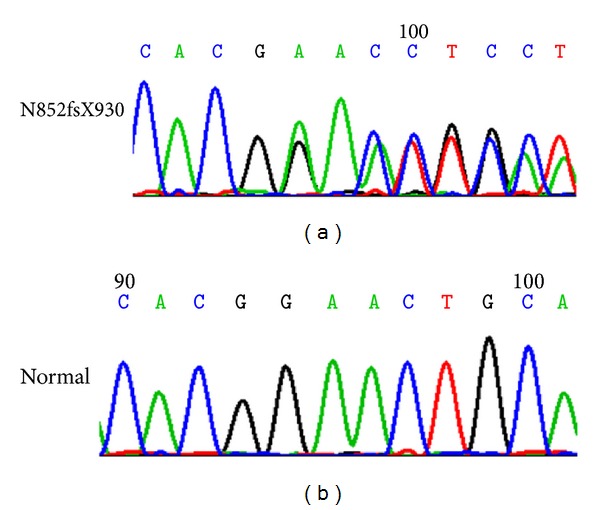
PKP2 mutation (above) versus wide type (bellow).

An 18-year-old Chinese man was sent to the hospital after suffering syncope while playing basketball. After a 12-lead ECG revealed ventricular tachycardia (Figure 1) and a subsequent 24 hr Holter monitor and echocardiography revealed suggestive changes, ARVC was diagnosed according to the 1994 task force criteria [5], and the patient underwent VT ablation. After discharge, episodes of palpitation and syncope persisted during physical activity despite ablation. ICD therapy was strongly recommended to the family who ultimately declined for financial reasons. Bisolol daily and amiodarone were administered with eventual discontinuation of the amiodarone after one year. Episodes of palpitation and syncope disappeared with medical therapy, but the patient suffered sudden cardiac death (SCD) three years later while swimming at 22 years of age. 12-lead ECGs, 24 hr Holter monitoring, and echocardiography were recorded yearly during the 5-year followup. 12-lead ECG showed a markedly fractionated QRS and terminal S wave prolongation. The QRS duration was 130 ms with a large epsilon wave (Figure 2). Holter ECG revealed frequent multifocal PVCs (n3000–4000/24 h) and nonsustained VT with left bundle branch block morphology. His electrocardiogram and echocardiography worsened gradually from the onset of syncopal episodes to death. (Figure 3, Table 1). Holter ECG revealed frequent multifocal PVCs and nonsustained VT (Figure 4). Genetic testing affirmed the diagnosis through the identification of a PKP2 mutation (Figure 5).

## 3. Discussion

The deterioration of parameters on ECG and ECHO indicates a poor prognosis. Such clearly illustrated progression from diagnosis to SCD on clinical testing allows for better visualization of disease progression and may heighten clinician suspicion of disease and recognition of an ominous course. The lifesaving therapy for ARVC is ICD placement and this case corroborates the diminishment of the prognosis in its absence.

## Figures and Tables

**Table 1:** Right ventricle size on echocardiography.

	2005	2006	2007	2008
RVIDd (mm)	45	48		48
RVD (mm)	17.5	26	32	35.7
RVOT (mm)	29		38	30
